# Neoadjuvant FOLFOXIRI plus bevacizumab without radiotherapy for high-risk rectal cancer: multicentre phase II trial

**DOI:** 10.1093/bjsopen/zraf163

**Published:** 2026-02-09

**Authors:** Takeru Matsuda, Yoshiaki Nagatani, Yohei Funakoshi, Takahiro Tsuboyama, Yasuhiko Mii, Kunihiko Kaneda, Tomohiro Tanaka, Hiroshi Hasegawa, Kimihiro Yamashita, Naomi Kiyota, Hironobu Minami, Yoshihiro Kakeji

**Affiliations:** Division of Gastrointestinal Surgery, Department of Surgery, Kobe University Graduate School of Medicine, Kobe, Japan; Department of Medical Oncology and Hematology, Kobe University Graduate School of Medicine, Kobe, Japan; Department of Medical Oncology and Hematology, Kobe University Graduate School of Medicine, Kobe, Japan; Department of Radiology, Kobe University Graduate School of Medicine, Kobe, Japan; Department of Surgery, Kitaharima Medical Centre, Ono, Japan; Department of Surgery, Kakogawa Central City Hospital, Kakogawa, Japan; Department of Surgery, Kakogawa Central City Hospital, Kakogawa, Japan; Division of Gastrointestinal Surgery, Department of Surgery, Kobe University Graduate School of Medicine, Kobe, Japan; Division of Gastrointestinal Surgery, Department of Surgery, Kobe University Graduate School of Medicine, Kobe, Japan; Department of Medical Oncology and Hematology, Kobe University Graduate School of Medicine, Kobe, Japan; Department of Medical Oncology and Hematology, Kobe University Graduate School of Medicine, Kobe, Japan; Division of Gastrointestinal Surgery, Department of Surgery, Kobe University Graduate School of Medicine, Kobe, Japan

## Abstract

**Background:**

The optimal neoadjuvant strategy for high-risk locally advanced rectal cancer (LARC) remains a matter of debate. This study evaluated the efficacy and safety of neoadjuvant FOLFOXIRI (fluorouracil, leucovorin, oxaliplatin, irinotecan) plus bevacizumab without radiotherapy in patients with magnetic resonance imaging-defined high-risk LARC.

**Methods:**

A prospective, multicentre, single arm phase II trial was conducted in four Japanese Institutions between 2018 and 2024, enrolling patients with rectal adenocarcinoma and at least one high-risk criterion: clinical T4, lateral pelvic lymph node metastasis, mesorectal fascia involvement, or positive extramural vascular invasion. Patients received four cycles of FOLFOXIRI plus bevacizumab, followed by two cycles of FOLFOXIRI alone, before total mesorectal excision. The primary endpoint was pathological complete response (pCR); secondary endpoints included the R0 resection rate, local recurrence (LR), recurrence-free survival (RFS), overall survival (OS), and safety.

**Results:**

OF 50 eligible patients, 31 were enrolled before early trial closure due to a slow accrual (accrual rate 62%). All patients underwent surgery. The pCR rate was 10% (3 of 31) and R0 resection was achieved in 97% (30 of 31) of patients. The median follow-up was 36.7 months. The 3-year cumulative LR rate was 3%, with 3-year RFS and OS rates of 73 and 81%, respectively. Grade ≥ 3 neutropenia occurred in 29% of patients, with acceptable toxicity overall. No cases of gastrointestinal perforation were observed. Grade ≥ III postoperative complications occurred in seven patients (23%), with the most frequent events being anastomotic leakage in two patients (7%).

**Conclusions:**

In this phase II trial, although recruitment was suboptimal, neoadjuvant FOLFOXIRI plus bevacizumab achieved good local control without radiotherapy in patients with high-risk LARC. Although the pCR rate was modest compared with radiotherapy-based regimens, this chemotherapy-only approach may represent a reasonable option for select patients who are not suitable candidates for pelvic radiotherapy. Registration number: UMIN000037367 (https://www.umin.ac.jp/english/).

## Teaser

This multicentre phase II study evaluated neoadjuvant FOLFOXIRI (fluorouracil, leucovorin, oxaliplatin, irinotecan) plus bevacizumab in patients with locally advanced low rectal cancer. Although the pathological complete response rate was modest, the regimen achieved favourable local control, with excellent R0 resection and low local recurrence rates, suggesting its potential efficacy in select high-risk patients.

## Introduction

Neoadjuvant chemoradiotherapy followed by total mesorectal excision (TME) has long been the standard treatment for locally advanced rectal cancer (LARC), providing improved local control but limited efficacy in reducing distant metastases^1–4^. Total neoadjuvant therapy (TNT) has recently shown promise in improving the pathological response and reducing systemic recurrence, becoming a widely accepted approach for high-risk patients^5,6^.

However, the use of radiotherapy could lead to potential long-term toxicity and technical challenges during surgery. As a result, there is growing interest in identifying patient subgroups in which radiotherapy could be safely omitted without compromising oncological outcomes. Recent trials, such as the PROSPECT study, demonstrated the non-inferiority of neoadjuvant chemotherapy (nCT) alone compared with neoadjuvant chemoradiotherapy in select patients^7^. Another phase II randomized trial (CAO/ARO/AIO 0214) reported pathological complete response (pCR) rates of 26 and 19% (*P* = 0.47) for patients treated with mFOLFOX6 (modified fluorouracil, leucovorin, oxaliplatin) and mFOLFOX6 plus aflibercept, respectively^8^. Nonetheless, these studies largely focused on low-risk LARC, and evidence in high-risk populations remains scarce.

FOLFOXIRI (fluorouracil, leucovorin, oxaliplatin, irinotecan) combined with bevacizumab has demonstrated high efficacy in metastatic colorectal cancer^9–13^, but its role in the neoadjuvant setting for high-risk LARC is not well established. The aim of this prospective multicentre phase II trial was to investigate the feasibility, efficacy, and safety of neoadjuvant FOLFOXIRI plus bevacizumab without radiotherapy in patients with magnetic resonance imaging (MRI)-defined high-risk LARC.

## Methods

### Study design and participants

This multicentre prospective open-label single-arm phase II trial of FOLFOXIRI plus bevacizumab was conducted at four institutions in Japan: Kobe University Hospital (Kobe, Hyogo Prefecture), Kitaharima Medical Center (Ono, Hyogo Prefecture), Kakogawa Central City Hospital (Kakogawa, Hyogo Prefecture), and Yodogawa Christian Hospital (Osaka, Osaka Prefecture). All participating hospitals are university-affiliated or major teaching hospitals performing approximately 50–150 rectal cancer surgeries per year. The study protocol was reviewed and approved by the Institutional Review Board and Ethics Committee of Kobe University Graduate School of Medicine (Approval no. B230160), as well as by the institutional review boards of all participating centres. The study adhered to the principles of the Declaration of Helsinki and Good Clinical Practice guidelines. Written informed consent was obtained from all patients before enrolment. The trial was registered with the University Hospital Medical Information Network (UMIN) Clinical Trials Registry (ID: UMIN000037367).

The eligibility criteria included: pathologically proven rectal adenocarcinoma; a distal border of the tumour located below the peritoneal reflection; a cT3 4N0 3M0 tumour stage according to the Japanese classification of colorectal, appendiceal, and anal carcinoma^14^; age 20–75 years; Eastern Cooperative Oncology Group performance status of 0 or 1; written informed consent; and preserved organ function. Patients were required to meet at least one high-risk criterion, namely: clinical T4 tumour; clinically positive lateral pelvic lymph node (LLN) metastasis (N3); mesorectal fascia (MRF) involvement (MRF margin ≤ 1 mm); and/or positive extramural vascular invasion (EMVI).

The exclusion criteria were: previous systemic chemotherapy, radiotherapy, hormone therapy, or immunotherapy treatment for the current disease; peripheral neuropathy of grade ≥ 1; uncontrolled hypertension; positive for hepatitis B surface antigen or anti-hepatitis C virus antibody; serious co-morbidities (heart failure, renal failure, liver failure, severe lung disease, haemorrhagic peptic ulcer, diarrhoea grade ≥ 2, uncontrolled diabetes); simultaneous multiple cancers or metachronous multiple cancers with a disease-free period of ≤ 5 years; pregnancy or breastfeeding; and homozygous for the UGT1A1*28 or *UGT1*A1*6 alleles of the UDP glucuronosyltransferase family 1 member A1 (*UGT1A1*) gene and heterozygous for the UGT1A1*6 and UGT1A1*28 alleles.

### Outcomes of interest

The primary endpoint was the pCR rate. Secondary endpoints included the R0 resection rate, postoperative complication rate, local recurrence (LR) rate, recurrence-free survival (RFS), overall survival (OS), and treatment-related safety.

RFS was defined as the time from surgery to the first documented recurrence or death from any cause. OS was defined as the time from surgery to death from any cause. Tumour response was evaluated using RECIST version 1.1^15^. Adverse events (AEs) were assessed by the investigators using the NCI Common Terminology Criteria for Adverse Events (CTCAE), version 4.0^16^.

### Pathological analysis

Tumour response to nCT was evaluated according to the grading system of the Japanese classification of colorectal, appendiceal, and anal carcinoma^14^, as follows: grade 0, no response; grade 1a, tumour reduction of one-third or less; grade 1b, tumour reduction greater than one-third and up to (and including) two-thirds; grade 2, a greater than two-thirds reduction in tumour size; and grade 3, pCR. Pathological assessment was performed independently at each participating institution without central pathology review.

### Treatment

After enrolment, patients received four cycles of FOLFOXIRI plus bevacizumab every 2 weeks. Each cycle included oxaliplatin (85 mg/m^2^), irinotecan (150 mg/m^2^), 5-fluorouracil (3200 mg/m^2^), leucovorin (200 mg/m^2^), and bevacizumab (5 mg/kg). After restaging with chest-to-pelvis computed tomography and colonoscopy, patients showing stable disease, a partial response, or a complete response (CR) received two additional cycles of FOLFOXIRI without bevacizumab. Patients with progressive disease or stable disease without evidence of tumour shrinkage were switched to salvage treatment including surgery. Prophylactic administration of PEGylated granulocyte colony-stimulating factor was allowed during nCT.

Surgery was performed 2–8 weeks after the final dose of 5-fluorouracil. The surgery consisted of TME with or without lateral pelvic lymph node dissection. Lateral pelvic lymph node dissection was performed in patients with radiologically suspected LLN metastasis, defined as a short-axis diameter ≥ 7 mm on computed tomography or MRI, a positive signal on diffusion-weighted MRI, or high uptake on positron emission tomography imaging. Combined resection of adjacent organs, including total pelvic exenteration, was allowed when necessary to achieve R0 resection. All surgeries were performed using minimally invasive approaches (laparoscopic or robotic); no open procedures were performed. The decision to use a transanal TME approach was made according to the surgeon’s discretion and institutional policy.

Adjuvant chemotherapy (CapeOX (capecitabine plus oxaliplatin) or FOLFOX6) was considered for all eligible patients irrespective of pathological stage, but was not part of the protocol.

AEs were monitored continuously during nCT and the perioperative period. Grading was based on CTCAE v4.0.

### Follow-up

Patients were followed every 3 months during the first 3 years and every 6 months thereafter. Tumour markers, including carcinoembryonic antigen and carbohydrate antigen 19-9, were assessed at each visit. Chest–pelvis computed tomography was performed every 6 months, and a total colonoscopy was conducted annually.

### Statistical analysis

The FOWARC trial^17^ reported a 6.6% pCR rate with neoadjuvant mFOLFOX6 in LARC, the N-SOG 03 phase II trial^18^ using CapeOX plus bevacizumab in high-risk LARC reported a 13% pCR rate, previous phase III trials^7,17,19^ using fluoropyrimidine-based chemoradiotherapy reported pCR rates ranging from 14 to 27.5%, and another phase II study of nCT with S-1 plus oxaliplatin in patients with LARC reported a pCR rate of 22.9%^20^. Based on these data, the expected and threshold pCR rates in the present study were set at 35 and 15%, respectively. The number of patients required was calculated with the exact binomial distribution method under the conditions of a two-sided significance level of 5% and a statistical power of 80%; based on these calculations, 36 patients needed to be recruited to the study. Allowing for a few dropouts or ineligible patients, the target sample size was set at 40.

The Kaplan–Meier method was used to estimate RFS and OS. All statistical analyses were performed using EZR (Saitama Medical Center, Jichi Medical University, Saitama, Japan), which is a graphical user interface for R (R Foundation for Statistical Computing, Vienna, Austria).

## Results

### Baseline patient characteristics

**Fig. 1:**
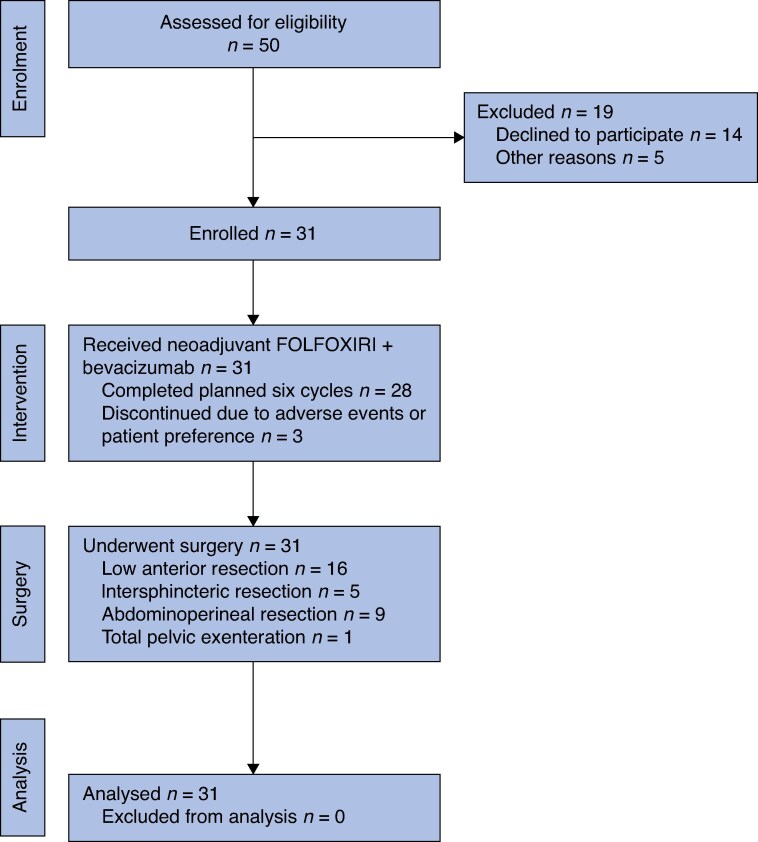
Consort diagram FOLFOXIRI, fluorouracil, leucovorin, oxaliplatin, irinotecan.

During the study period, 50 patients were deemed eligible for inclusion in the study. Of these patients, 31 were enrolled between March 2018 and March 2024 (accrual rate 62%; *Fig. 1*). The trial was terminated early due to slow accrual.

Baseline patient and tumour characteristics are summarized in *Table 1*. The median tumour distance from the anal verge was 3.0 cm. Among the 31 patients, 16 (52%) had cT4a or cT4b tumours and 16 (52%) had cN3 disease (LLN involvement). MRF involvement and positive EMVI on pretreatment MRI were observed in 21 patients (68%) and 10 patients (32%), respectively. All patients met at least one high-risk criterion: cT4, cN3, involvement of MRF, or positive EMVI.

**Table 1:** Baseline demographic and clinical characteristics for the total cohort (*n* = 31)

Age (years), median (i.q.r.)	58 (52–65)
**Sex**	
Male	23 (74%)
Female	8 (26%)
Body mass index (kg/m^2^), median (i.q.r.)	24.0 (21.5–26.0)
**ECOG performance status**	
0	23 (74%)
1	8 (26%)
Pretreatment CEA (ng/ml), median (i.q.r.)	6.9 (2.1–25.0)
Pretreatment CA19-9 (U/ml), median (i.q.r.)	17.0 (5.5–43.6)
Distance from anal verge (cm), median (i.q.r.)	3.0 (1.0–6.0)
cT*	
1/2	0
3	15 (48%)
4a	12 (39%)
b	4 (13%)
cN*	
0	3 (10%)
1	6 (19%)
2	6 (19%)
3	16 (52%)
cStage*	
II	3 (10%)
IIIa	1 (3%)
IIIb	13 (42%)
IIIc	14 (45%)
**High-risk features**	
Lateral lymph node metastasis (cN3)	16 (52%)
cT4 tumour	14 (45%)
MRF involved	21 (68%)
Positive for EMVI	10 (32%)
***UGT1A1* status**	
Wild-type	23 (74%)
Heterozygous (*6 or *28)	8 (26%)

Values are *n* (%) unless otherwise stated. *According to the Japanese classification of colorectal, appendiceal, and anal carcinoma^14^. i.q.r., interquartile range; ECOG, Eastern Cooperative Oncology Group; CEA, carcinoembryonic antigen; CA19-9, carbohydrate antigen 19-9; MRF, mesorectal fascia; EMVI, extramural vascular invasion; *UGT1A1*, UDP glucuronosyltransferase family 1 member A1.

### nCT adherence and toxicity

In all, 28 patients (90%) received all six planned cycles of FOLFOXIRI, and 29 patients (94%) received all four cycles of bevacizumab (*Fig. 1*). The median number of cycles administered was six for FOLFOXIRI and four for bevacizumab (*Table 2*). A dose reduction of any drug was required in 17 patients (55%), primarily due to toxicity. Reductions occurred at similar rates for 5-fluorouracil, oxaliplatin, and irinotecan.

**Table 2:** Operative and postoperative outcomes for the entire cohort (*n* = 31)

**Operative procedure**	
Low anterior resection	16 (52%)
Intersphincteric resection	5 (16%)
Abdominoperineal resection	9 (29%)
Total pelvic exenteration	1 (3%)
**Combined resection**	
Prostate	1 (3%)
Vagina	1 (3%)
Ureter	1 (3%)
Bladder	1 (3%)
None	28 (90%)
Lymph node dissection*	
prxD2	3 (10%)
prxD3	28 (90%)
**LLND**	
Unilateral	13 (42%)
Bilateral	7 (23%)
None	11 (36%)
**Approach**	
Open	0
Laparoscopic	21 (68%)
Robotic	10 (32%)
**TaTME utilization**	
Yes	26 (84%)
No	5 (16%)
Operation time (min), median (i.q.r.)	328 (255–488)
Estimated blood loss (g), median (i.q.r.)	20 (0–95)
**Transfusion**	
Yes	2 (7%)
No	29 (94%)
**Resection status**	
R0	30 (97%)
R1	1 (3%)
**Postoperative complications**	
CD grade ≥II	12 (39%)
CD grade ≥III	7 (23%)
30-day mortality	0
30-day reoperation	0
Postoperative LOS (days), median (i.q.r.)	17 (14–24)
Adjuvant chemotherapy	22 (71%)

Values are *n* (%) unless otherwise stated. *According to the Japanese classification of colorectal, appendiceal, and anal carcinoma^14^. LLND, lateral pelvic lymph node dissection; TaTME, transanal total mesorectal excision; min, minutes; i.q.r., interquartile range; CD, Clavien–Dindo; LOS, length of hospital stay.

All patients experienced at least one AE of any grade. As indicated in *Table S1*, the most common AEs of any grade were anorexia (68%), fatigue (68%), peripheral neuropathy (58%), and anaemia (55%). The most frequent grade ≥ 3 AE was neutropenia, observed in 29% of patients. Three patients (10%) received prophylactic administration of PEGylated granulocyte colony-stimulating factor.

### Surgical outcomes

There were no patients with progressive disease or no tumour shrinkage after restaging, and all patients underwent surgery following nCT. Among the 31 patients, 26 received transanal TME. One patient underwent total pelvic exenteration (*Table 3*). Lateral pelvic lymph node dissection, either unilateral or bilateral, was performed in 20 patients (65%).

**Table 3:** Compliance with neoadjuvant chemotherapy across the entire cohort (*n* = 31)

Total no. of FOLFOXIRI cycles, median (i.q.r.)	6 (6–6)
Total no. of bevacizumab cycles, median (i.q.r.)	4 (4–4)
**Dose reduction of chemotherapy agents**	
5-FU	15 (48%)
Oxaliplatin	13 (42%)
Irinotecan	16 (52%)
Bevacizumab	0
**Discontinuation of chemotherapy**	
FOLFOXIRI	3 (10%)
Bevacizumab	2 (7%)

Values are *n* (%) unless otherwise stated. FOLFOXIRI, fluorouracil, leucovorin, oxaliplatin, irinotecan; i.q.r., interquartile range; 5-FU, 5-fluorouracil.

Only one patient, who underwent intersphincteric resection, had an R1 resection.

Postoperative complications of grade ≥ II and III according to the Clavien–Dindo classification^21^ developed in 12 patients (39%) and 7 patients (23%), respectively (*Table S2*). The most common complications were urinary disturbance (grade II) in two patients (7%) and anastomotic leakage (grade IIIb) in two patients (7%).

### Pathological findings

Pathological outcomes are summarized in *Table 4*. Four patients (13%) had yp (post-therapy) T0 or ypTis tumours, and the pCR rate was 10% (3 of 31 patients). Two patients (7%) had ypT4 tumours, nine (29%) had residual nodal disease (ypN+), and none had pathologically confirmed LLN metastasis. Positive distal or circumferential resection margins (≤ 1 mm) were observed in one patient who underwent intersphincteric resection, corresponding to the only R1 resection in the study. The R0 resection rate was 97% (30 of 31 patients). A good pathological response to nCT (grade 2 or 3) was observed in 12 patients (39%).

**Table 4:** Pathological outcomes in the total cohort (*n* = 31)

**Histological type**	
Differentiated	28 (90%)
Undifferentiated	3 (10%)
ypT*	
0/is	4 (13%)
1	1 (3%)
2	6 (19%)
3	18 (58%)
4a	1 (3%)
4b	1 (3%)
ypN*	
0	22 (71%)
1	6 (19%)
2	3 (10%)
3	0
ypStage*	
0	4 (13%)
I	7 (23%)
II	11 (36%)
IIIa	1 (3%)
IIIb	5 (16%)
IIIc	3 (10%)
Positive LLN metastasis	0
**Lymphovascular invasion**	
Absent	22 (71%)
Present	9 (29%)
**Circumferential margin**	
Negative (> 1 mm)	30 (97%)
Positive (≤ 1 mm)	1 (3%)
**Distal margin**	
Negative (> 1 mm)	30 (97%)
Positive (≤ 1 mm)	1 (3%)
**Resection status**	
R0	30 (97%)
R1	1 (3%)
Pathological response*	
Poor (Grade 0, 1a, 1b)	19 (61%)
Good (Grade 2, 3)	12 (39%)
Pathological complete response	3 (10%)

Values are *n* (%). *According to the Japanese classification of colorectal, appendiceal, and anal carcinoma^14^. yp, post-therapy; LLN, lateral pelvic lymph node.

### Survival and LR

**Fig. 2:**
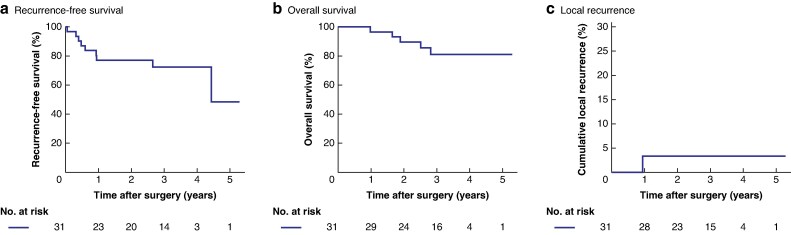
Kaplan–Meier curves of recurrence-free survival, overall survival, and the cumulative incidence of local recurrence after surgery **a** Recurrence-free survival. At 3 years, the recurrence-free survival rate was 78% (95% c.i. 52 to 85%). **b** Overall survival. At 3 years, the overall survival rate was 81% (95% c.i. 60 to 92%). **c** Cumulative incidence of local recurrence after surgery. The local recurrence rate at 3 years was 3% (95% c.i. 0.5 to 21%). c.i., confidence interval.

The median follow-up duration was 36.7 months (interquartile range 28–44 months). The 3-year RFS and OS rates were 73% (95% confidence interval (c.i.) 52 to 85%) and 81% (95% c.i. 60 to 92%), respectively (*Fig. 2*).

Only one patient, who underwent intersphincteric resection, developed LR at the anastomotic site, resulting in a 3-year cumulative LR rate of 3% (95% c.i. 0.5 to 21%).

The most common site of recurrence was the lung (4 patients, 13%). Other recurrence sites included the brain, liver, multiple lymph nodes, and peritoneum.

## Discussion

This prospective multicentre phase II trial evaluated the safety and efficacy of neoadjuvant FOLFOXIRI plus bevacizumab without radiotherapy in patients with MRI-defined high-risk LARC. Although the pCR rate was modest at 10%, the regimen achieved a high R0 resection rate (97%) and excellent local control, with a 3-year cumulative LR rate of only 3%. These findings suggest that a systemic triplet regimen with anti-angiogenic therapy may offer a viable alternative to radiotherapy-containing strategies in select high-risk patients, particularly where long-term radiotherapy toxicity or surgical complexity is a concern. Notably, despite the aggressive tumour characteristics of the cohort (for example, 52% cT4, 68% MRF involvement), surgical feasibility and adherence to chemotherapy were preserved.

Although the pCR rate in this study was lower than anticipated, this may be partly attributed to the inclusion of patients with high-risk features, such as cT4 tumours, clinically positive LLN metastasis, positive EMVI, and MRF involvement. The PROSPECT trial, which included patients with LARC clinically staged as T2 node-positive, T3 node-negative, or T3 node-positive who were candidates for sphincter-sparing surgery, revealed that 117 of 535 patients (21.9%) in the FOLFOX group achieved pCR, although 53 (9.1%) of these patients received additional neoadjuvant chemoradiotherapy^7^ . The CAO/ARO/AIO 0214 trial, which investigated mFOLFOX6 with and without aflibercept in patients with LARC staged T3 with negative MRI-predicted circumferential resection margins who were candidates for sphincter-sparing surgery, reported a pCR rate of 26% in the mFOLFOX6 alone group^8^. In contrast, the present study specifically targeted a high-risk cohort, in which tumour regression is generally more difficult to achieve. Nevertheless, the oncological outcomes in terms of local control and R0 resection suggest that pCR may not be the most appropriate or sufficient endpoint for this patient population.

Compared with recent TNT trials, such as RAPIDO^5^ and STELLAR^22^, the LR rate in the present study was notably low. These previous trials, while reported higher pCR rates, had 3-year LR rates of approximately 8%, even with the use of radiotherapy^5,22^. Moreover, challenges in achieving R0 resection following TNT due to treatment-related tissue changes have been reported^23^. In contrast, the approach used in the present study achieved an R0 resection rate of 97%, highlighting the potential of systemic chemotherapy alone to achieve local tumour control without compromising surgical quality.

Although this trial demonstrated favourable local control and high R0 resection rates, the pCR rate of 10% was lower than that reported in recent TNT trials, such as RAPIDO^5^ and PRODIGE 23^6^. This difference highlights that, although intensive systemic chemotherapy may contribute to tumour downstaging and excellent local control, it may not achieve the same degree of tumour regression as radiotherapy-containing regimens. Therefore, the results of the present study should not be interpreted as evidence that chemotherapy-only strategies can replace standard TNT in routine practice. Rather, they suggest that this regimen may represent a reasonable option in select patients who are ineligible for, or who decline, pelvic radiotherapy (for example, those with a history of previous pelvic irradiation, active inflammatory bowel disease, or significant concern about long-term radiation toxicity). Future randomized comparative studies are warranted to clarify whether systemic chemotherapy alone can achieve durable local control comparable to radiation-based approaches in specific subgroups of patients with high-risk rectal cancer.

The regimen was well tolerated overall. Most patients completed the planned six cycles of FOLFOXIRI, and AEs were manageable. The use of bevacizumab raised no significant concerns regarding wound healing or bleeding complications, and no cases of gastrointestinal perforation were reported. All patients underwent surgery after nCT. The rate of grade ≥ III postoperative complications in this study (23%) was still suboptimal, although previous TNT or chemoradiotherapy trials, such as RAPIDO^5^ and PRODIGE 23^6^, reported comparable incidences (20–25%) of major complications, mainly anastomotic leakage or pelvic sepsis. These findings suggest that preoperative FOLFOXIRI plus bevacizumab did not increase postoperative morbidity and may be considered surgically safe when an appropriate interval is maintained between chemotherapy and surgery.

However, according to recent studies^8,24,25^, the addition of the anti-angiogenic agent to nCT or TNT seems not to be recommended due to higher AE rates and no benefit in oncological outcomes. Therefore, the effect of adding bevacizumab to nCT or TNT needs to be further investigated in future prospective comparative studies.

This study has several limitations. First, it was a small-scale phase II study, and the target sample size was not achieved. However, the statistical power was 75%. In addition, the diagnosis of LLN metastasis was based on institutional radiological assessment, which may have resulted in overstaging in some cases. A central imaging review could enhance future study accuracy. Finally, the absence of a comparator arm precludes definitive conclusions regarding the superiority or equivalence of this approach to standard chemoradiotherapy or TNT.

Overall, neoadjuvant FOLFOXIRI plus bevacizumab demonstrated excellent local control and high R0 resection rates in patients with MRI-defined high-risk rectal cancer, despite a modest pCR rate. This approach may be considered for select patients who are not candidates for pelvic radiotherapy. Further studies are needed to confirm its oncological efficacy and define its optimal role within multimodal treatment strategies.

## Supplementary Material

zraf163_Supplementary_Data

## Data Availability

The data used for the analysis are restricted-use data from Kobe University Hospital, and can only be accessed by researchers with signed data use agreements.
