# Supplementary material for: Neoadjuvant FOLFOXIRI plus bevacizumab without radiotherapy for high-risk rectal cancer: multicentre phase II trial
Source: BJS Open. 2026 Feb 9;10(1):zraf163. doi: 10.1093/bjsopen/zraf163 (PMC12884854; doi:10.1093/bjsopen/zraf163)
Supplement: zraf163_Supplementary_Data [file zraf163_supplementary_data.docx]

**Neoadjuvant FOLFOXIRI Plus Bevacizumab Without Radiotherapy for High-Risk Rectal Cancer: A Multicentre Phase II Trial**

Authors

**Takeru Matsuda, MD, PhD,^1^ Yoshiaki Nagatani, MD, PhD,^2^ Yohei Funakoshi, MD, PhD,^2^ Takahiro Tsuboyama, MD, PhD,^3^ Yasuhiko Mii, MD, PhD,^4^ Kunihiko Kaneda, MD, PhD,^5^ Tomohiro Tanaka, MD, PhD,^5^ Hiroshi Hasegawa, MD, PhD,^1^ Kimihiro Yamashita, MD, PhD,^1^ Naomi Kiyota, MD, PhD,^2^ Hironobu Minami, MD, PhD,^2^ Yoshihiro Kakeji, MD, PhD^1^**

^1^Division of Gastrointestinal Surgery, Department of Surgery, Kobe University Graduate School of Medicine, Kobe, Japan

^2^Department of Medical Oncology and Hematology, Kobe University Graduate School of Medicine, Kobe, Japan.

^3^Department of Radiology, Kobe University Graduate School of Medicine, Kobe, Japan.

^4^Department of Surgery, Kitaharima Medical Centre, Ono, Japan

^5^Department of Surgery, Kakogawa Central City Hospital, Kakogawa, Japan

**Corresponding author.** Takeru Matsuda, M.D., Ph.D.

Division of Gastrointestinal Surgery, Department of Surgery, Kobe University Graduate School of Medicine

7-5-2 Kusunoki-chou, Chuo-ku, Kobe 650-0017, JAPAN

Tel: +81-78-382-5925 Fax: +81-78-382-5939

E-mail: [takerumatsuda@nifty.com](mailto:takerumatsuda@nifty.com)

**Supplementary Materials - Index**

| **Supplementary Tables** |  |
| --- | --- |
| **Supplementary Table 1** | *Table S1 pag. 2* |
| **Supplementary Table 2** | *Table S2 pag. 3* |

**Table S1. Adverse events with neoadjuvant chemotherapy (n = 31)**

| **Adverse event, n (%)** | **Grade 1-2** | **Grade 3** | **Grade 4** |
| --- | --- | --- | --- |
| Leukemia | 5 (16.1) | 1 (3.2) | 0 |
| Neutropenia | 4 (12.9) | 8 (25.8) | 1 (3.2) |
| Febrile neutropenia | 0 | 0 | 0 |
| Thrombocytopenia | 7 (22.6) | 0 | 0 |
| Anemia | 17 (54.8) | 0 | 0 |
| Increased AST/ALT | 3 (9.7) | 0 | 0 |
| Increased creatinine | 1 (3.2) | 0 | 0 |
| Nausea | 16 (51.6) | 0 | 0 |
| Vomiting | 2 (6.5) | 2 (6.5) | 0 |
| Anorexia | 18 (58.1) | 3 (9.7) | 0 |
| Diarrhea | 8 (25.8) | 5 (16.1) | 0 |
| Constipation | 7 (22.6) | 0 | 0 |
| Allergic reaction | 0 | 0 | 0 |
| Fatigue | 18 (58.1) | 3 (9.7) | 0 |
| Hand-foot syndrome | 2 (6.5) | 0 | 0 |
| Peripheral neuropathy | 18 (58.1) | 0 | 0 |
| Oral mucositis | 3 (9.7) | 1 (3.2) | 0 |
| Dysgeusia | 3 (9.7) | 0 | 0 |
| Cholinergic syndrome | 1 (3.2) | 1 (3.2) | 0 |
| Insomnia | 1 (3.2) | 0 | 0 |
| Alopecia | 13 (41.9) | 0 | 0 |
| Epistaxis | 1 (3.2) | 0 | 0 |
| Hiccups | 2 (6.5) | 0 | 0 |
| Hypertension | 1 (3.2) | 1 (3.2) | 0 |
| Gastrointestinal perforation | 0 | 0 | 0 |
| Rectal bleeding | 1 (3.2) | 0 | 0 |

**Table S2. Postoperative complications (CD grade ≥ II)**

| **Postoperative complication, n (%)** | **Grade II** | **Grade IIIa** | **Grade IIIb** |
| --- | --- | --- | --- |
| Urinary disturbance | 2 (6.5) | 0 | 0 |
| Anastomotic leakage | 0 | 0 | 2 (6.5) |
| Abdominal wound infection | 0 | 0 | 0 |
| Perineal wound infection | 0 | 1 (3.2) | 0 |
| Pelvic infection | 1 (3.2) | 0 | 0 |
| Bowel obstruction | 0 | 0 | 1 (3.2) |
| Paralytic ileus | 0 | 0 | 0 |
| Lymphorrhea | 0 | 1 (3.2) | 0 |
| Ureteral injury | 0 | 1 (3.2) | 0 |
| Bleeding | 1 (3.2) | 1 (3.2) | 0 |
| Pneumonia | 0 | 0 | 0 |
| Others | 2 (6.5) | 0 | 0 |

*CD* Clavien-Dindo classification
